# 

**Published:** 1904-07

**Authors:** 


					﻿CONTENTS.
ORIGINAL COMMUNICATIONS—
Study of a Case of Lateral Curvature of the Spine. A Report on an Operation for theTe-
formity. By Michael Hoke, M.D., Atlanta, Ga...........................  217	,
Some Aspects of Medical Education. By John H. Musser, M.D., Philadelphia, Pa. ..72381
EDITORIAL NOTES AND COMMENTS—
Syphilis and Life-Insurance.............................................260
Hari-Kari ,..........................................................  f.	261 j
Notice................................................................. ... 26? I
NEWS AND'NOTES........................................................-.... 26S
CONTENTS CONTINUED........................................................... x
X	'	’	I
CONTENTS— CONTINUED.
BOOK REVIEWS—
Progressive Medicine............................................. 266
SELECTIONS AND ABSTRACTS—
Communities Without Health Departments in the Crusade Against Tuberculosis 267
Irregular Menstruation and Treatment............................. 278
Another Crusade.................................................. 281
Poisoning by the White of an Egg................................. 283
The History of Appendicitis..................................... 284.
Expert Witnesses Appointed by the Court...........................284
Drug Headaches................................................... 285
Lay Comments on a Lithopiedion ................................. 287
A Substitute for Rubber Gloves.................................. 28i
The Koreans.......................................................286
MEDICAL ITEMS—Continued.......................xviii,	xx, xxii, xxiv, xxvi, xxviii.
......
				

